# Utilizing Short Interspersed Nuclear Element as a Genetic Marker for Pre-Harvest Sprouting in Wheat

**DOI:** 10.3390/plants13212981

**Published:** 2024-10-25

**Authors:** Purnima Kandpal, Karminderbir Kaur, Raman Dhariwal, Simranjeet Kaur, Gagandeep Kaur Brar, Harpinder Randhawa, Jaswinder Singh

**Affiliations:** 1 Department of Plant Science, McGill University, 21111 Rue Lakeshore, Montreal, QC H9X 3V9, Canada; purnima.kandpal@mail.mcgill.ca (P.K.); karminderbir.kaur@mcgill.ca (K.K.); 2 Lethbridge Research and Development Centre, Agriculture and Agri-Food Canada, 5403 1st Avenue South, Lethbridge, AB T1J 4B1, Canada; raman.dhariwal@agr.gc.ca (R.D.); simranjeet.kaur@agr.gc.ca (S.K.); gagandeep.brar@agr.gc.ca (G.K.B.); harpinder.randhawa@agr.gc.ca (H.R.)

**Keywords:** pre-harvest sprouting, Argonaute, SINE, epigenetics, dormancy, DNA methylation, genetic marker

## Abstract

Pre-harvest sprouting (PHS) is a complex abiotic stress caused by multiple exogenous and endogenous variables that results in random but significant quality and yield loss at the terminal crop stage in more than half of the wheat-producing areas of the world. Systematic research over more than five decades suggests that addressing this challenge requires tools beyond the traditional genetic manipulation approach. Previous molecular studies indicate a possible role of epigenetics in the regulation of seed dormancy and PHS in crops, especially through RNA-directed DNA methylation (RdDM) pathways mediated by Argonaute (AGO) proteins. In this study, we explore the role of the *AGO802B* gene associated with PHS resistance in wheat, through the presence of a SINE retrotransposon insertion. The current study found the SINE insertion at 3′UTR of the *TaAGO802B* present in 73.2% of 41 cultivars analyzed and in 92.6% of the resistant cultivar subset. The average expression of *TaAGO802B* in cultivars with the SINE insertion was 73.3% lower than in cultivars without insertion. This study also indicated a significant positive correlation between the PHS score and methylation levels in the cultivars. The resistant cultivars with the SINE insertion recorded 54.7% lower methylation levels than susceptible cultivars. Further analysis of a DH population (Sadash × P2711) reveals that SINE insertion co-segregates with PHS resistance. This sets forth the SINE insertion in *TaAGO802B* as a genetic marker for screening wheat germplasm and as an efficient tool for breeding PHS-resistant wheat cultivars.

## 1. Introduction

Wheat ranks second after maize in terms of total crop yield. In total 808 million tons were produced, at an average yield of 3.69 t/ha, during 2022 [1], against the crop potential of up to 13 t/ha [2]. This gap in realized yield potential is attributed to several abiotic and biotic stresses affecting different stages of crop production [3]. Pre-harvest sprouting (PHS), one of the primary abiotic factors impacting the yield and quality of grains at the terminal crop stage is prevalent in more than half of the wheat-producing areas of the world [4] and results in an annual loss of more than USD 1 billion globally [5]. PHS is one of the major detrimental factors affecting the Canadian wheat economy, restricting the average productivity to just 3.40 t/ha, almost 8% lower than the world average and at least 43% lower than the comparable European agro-ecologies [1]. This results in annual losses of around USD 100 million for the Canadian wheat industry [6].

PHS in wheat was unknowingly but gradually accrued during domestication, mainly through modern wheat breeding programs [7]. As a result, most wheat cultivars, particularly the modern semi-dwarf varieties, are highly susceptible to PHS [8]. PHS refers to the germination of physiologically mature grains while still present on the parent plant upon prolonged exposure to wet weather conditions before harvest [9]. This ultimately leads to significant losses in yield, decreased falling number values, and substandard baking quality of the dough. Traditionally, the occurrence of PHS is sporadic, but the rapidly changing climate indicates a future with more frequent and intense PHS events [10]. Additionally, the increased demand for safe and assured food for a growing population and the current global trade scenario necessitate the mitigation of PHS-driven losses in wheat.

Systematic research efforts to address the challenge of PHS stress were initiated in the early nineteen-seventies. It was observed that this complex trait is mainly controlled by seed dormancy but is significantly influenced by environmental cues or abiotic stresses, viz., rainfall and temperature [11]; morphological traits, viz., awn length, head angle, spike shape, glume tenacity, waxy spikes, and seed color [12,13]; and physiological and biochemical factors [14]. The untimely rains during the harvest season increase the activity of amylases, lipases, and proteases in drying seeds, leading to the breakdown of stored starch, lipids, and proteins [12]. This process in seeds is mainly mediated by modulating the balance between plant growth regulators Abscisic acid (ABA) and Gibberellic acid (GA) [15].

Numerous quantitative trait loci (QTLs) associated with PHS have been reported throughout the wheat genome [16]. In total, 66 meta-QTLs have been identified across 21 wheat chromosomes [16], with particular attention given to QTLs on chromosomes 3AS, 4AL, and 2BS [17]. Recently, Dhariwal et al. identified seven major loci (on chromosomes 1A, 2B, 3A, 3B, 3D, and 7D), each explaining at least 10 percent of phenotypic variation for PHS resistance in wheat [6]. In addition to QTLs, seven candidate genes (*TaMFT/TaPHS1*, *Tamyb10*, *TaMKK*, *TaVp-1*, *TaDOG1*, *TaSdr*, *TaQsd1*) [12] associated with PHS have also been isolated through homology-based cloning and mapping, and four genes (*TaGASR34*, *TaVQ*, *TaCDPK21*, *TaCPK40*) have been identified using bioinformatics and transcriptomic tools [5]. These QTLs/genes primarily regulate seed dormancy by influencing the ABA/GA signaling pathways. However, their response varies with varying genetic backgrounds [5,17], changes in allele frequencies [18], and pleiotropic effects [19]. This suggests that the solution to the complex PHS problem may lie beyond the genetic manipulation of loci controlling hormonal balances or pathways specific to seed dormancy [20].

Recent advances in PHS research have focused on delineating the role of epigenetic modifications in the seed development and dormancy [7]. Accurate DNA processing and subsequent gene transcript levels have shown tight linkage to chromatin status, which is regulated by epigenetic modifications, including DNA methylation, histone modifications, chromatin remodeling, and the activity of small RNAs [21]. Epigenetic research in barley [22] and wheat [23] has indicated the involvement of *ARGONAUTE (AGO)* genes in gene silencing via DNA methylation. *AGO1003*, an *AGO4_9* gene in barley exhibits differential expression in the embryos of dormant and non-dormant cultivars. *AGO* genes are believed to act as negative regulators of seed dormancy through the RNA-dependent DNA methylation (RdDM) pathway [22]. Furthermore, the degree of PHS resistance in wheat cultivars was affected by the presence of short interspersed nuclear element (SINE) at 3′UTR of the *TaAGO802B*, an ortholog of barley *AGO1003* in wheat [23]. This 160bp insertion had 9bp repeats at both ends. This suggested a possible role of the *AGO* gene and SINE insertion in modulating PHS; SINE can be harnessed as a molecular marker for screening wheat germplasm against PHS if confirmed in genetically varied populations. We hypothesize that wheat germplasm exhibits genetic variability for SINE insertion in tandem with PHS response. Therefore, the present study was conducted to test this hypothesis across Canadian wheat cultivars and breeding lines of diverse genetic backgrounds to delve deeper into the role of *AGO* genes and SINE insertion in regulating PHS in wheat.

## 2. Results

### 2.1. PHS Phenotyping

**Figure 1:**
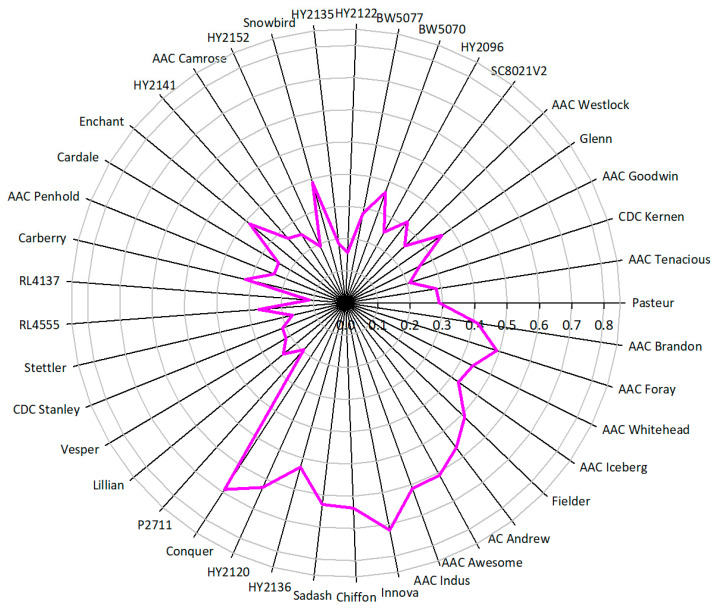
PHS score levels in diverse Canadian wheat germplasm. The pink line represents the distribution of scores on the scale represented by the radius. A higher score denotes high susceptibility to PHS, while values approaching 0 reflect increasing resistance.

The PHS assay revealed a significant variation in the PHS tolerance levels of the Canadian wheat cultivars analyzed (Figure 1). The PHS score varies between 0.102 and 0.718 on a zero-to-one scale among the evaluated cultivars, where a perfect score of one indicates highly susceptible cultivars and values approaching zero indicate an increasing level of PHS resistance. Of the 41 cultivars evaluated, 34.1% of cultivars were found to be moderately to highly susceptible to PHS with an average score of 0.548 (range: 0.411–0.718), and 65.9% of cultivars were resistant to PHS with an average score of 0.249 (range: 0.113–0.388), which is 54.6% lower than the average score of susceptible cultivars. Overall, cv. RL4137 was observed as the most tolerant to PHS, followed by HY2122. Contrastingly, cv. AAC Innova, Chiffon, and Conquer were found to be highly susceptible to PHS.

### 2.2. Polymorphism in Wheat Cultivars for SINE

**Figure 2:**
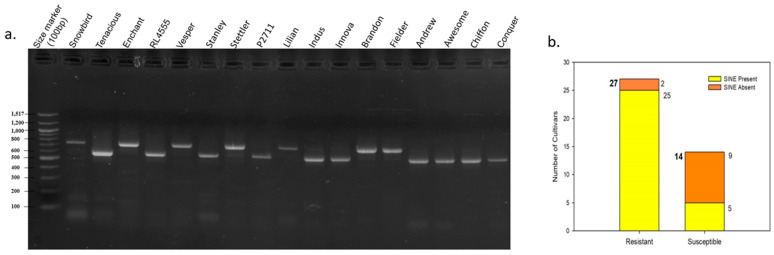
Analysis of SINE insertion in *AGO802B.* (**a**) Agarose gel displaying polymorphism in a subset of wheat cultivars. (**b**) Graphical representation of the association of SINE insertion with PHS tolerance in Canadian wheat cultivars.

The amplification of *AGO802B* in the current panel exhibited size polymorphism. A 160bp insertion in the 3′UTR, located 30bp downstream of the stop codon, was observed in various cultivars, viz., Enchant, Vesper, CDC Stanley, and AAC Penhold, but was absent in other cultivars, viz., AC Andrew, AAC Awesome, and Sadash (Figure 2a, Table 1). The presence of a 9bp duplication at both ends of the sequence in question suggests a retroelement origin, likely representing short interspersed nuclear element (SINE). Overall, 73.2% of cultivars contained the SINE insertion, but the pattern was highly skewed among the resistant and susceptible cultivars. Almost 92.6% of resistant cultivars had SINE insertion, while only 35.7% of susceptible cultivars had this insertion (Figure 2b). Among resistant cultivars, only two highly resistant cultivars, namely AAC Tenacious and Pasteur, lacked SINE insertion. Further association analysis revealed that the presence of SINE insertion indicates a 92.6% chance of a cultivar being resistant, and in contrast, the absence of the insertion indicates an 81.8% chance of a cultivar being susceptible to PHS.

A study of pangenome data also confirmed the polymorphism for this insertion in the wider wheat germplasm. Out of the 16 genome assemblies examined, 3 genomes, namely Landmark, Lancer, and Kariega, had the insertion (Supplementary Figure S1 and Supplementary Table S1). However, information on the level of PHS tolerance of these cultivars is needed for correlation with the current findings.

### 2.3. Gene Expression

**Figure 3:**
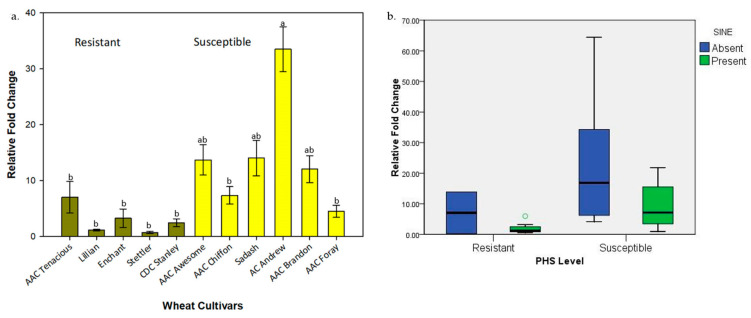
Expression of *AGO802* gene in various wheat cultivars. (**a**) Relative fold change in various wheat cultivars with varying levels of PHS. The lowercase letters above the bar indicate a significant difference (*p* ≤ 0.05). (**b**) Boxplot distribution of SINE insertion in PHS-resistant and PHS-susceptible wheat cultivars.

Relative fold change analysis revealed a consistent pattern of significant variation among the wheat cultivars for the expression of *TaAGO802*. Overall, susceptible cultivars, particularly, AAC Awesome, AAC Chiffon, and Sadash displayed markedly higher expression levels (average fold change value = 14.21). In comparison, resistant cultivars, viz., Enchant, Stettler, and CDC Stanley, had a 79.3% lower average expression (Figure 3a). Interestingly, the resistant cultivar AAC Tenacious exhibited an unexpectedly higher expression level compared to all other resistant cultivars analyzed, as well as one of the susceptible cultivars, AAC Foray, and showed expression levels comparable to another susceptible cultivar, AAC Chiffon.

The presence of SINE insertion was observed to have a significant impact on the expression of *TaAGO802*. In general, the presence of SINE insertion in a cultivar reduced the expression of *TaAGO802* by 73.3%, compared to the cultivars with no SINE insertion. However, this reduction in the average expression of *TaAGO802* was 72.6% among resistant cultivars but only 51.6% among susceptible cultivars (Figure 3b).

### 2.4. DNA Methylation Study

**Figure 4:**
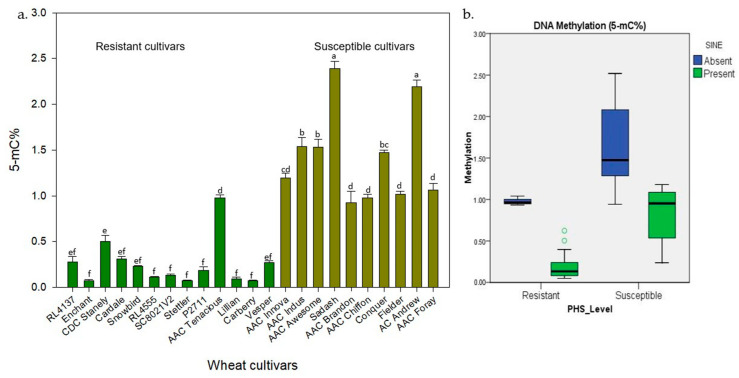
The study of global 5-mC% methylation levels in Canadian wheat cultivar panel: (**a**) 5-mC% in wheat cultivars having different levels of PHS. The lowercase letter above the bar indicates the significant difference (*p* ≤ 0.05). (**b**) Boxplot distribution of SINE insertion in PHS-resistant and susceptible wheat cultivars.

Global 5-mC% methylation levels of the 23 wheat genotypes ranged from 0.074 to 2.391, with an average of 0.77% (Figure 4a). PHS-resistant cultivars demonstrated lower levels of methylation, with values ranging from 0.27% to 0.97%, while PHS-susceptible cultivars displayed higher methylation levels on average, ranging from 0.98% to 2.39%. The average methylation level was 1.43% among the susceptible cultivars; however, resistant cultivars had 86.5% lower methylation levels than the susceptible cultivars. The correlation between the PHS score and the methylation level was found significant for the evaluated cultivars (r = 0.774). Interestingly, AAC Tenacious presented an unexpectedly higher methylation level, even when compared to AAC Brandon, a susceptible cultivar.

The presence of SINE insertion also affects the PHS level among the wheat cultivars. The cultivars with SINE insertion had a 54.7% lower average methylation level than cultivars with no SINE insertion. However, the methylation rate was on par among the susceptible and resistant cultivars with SINE insertion (Figure 4b).

### 2.5. Population Study

Of the 140 DH lines (Sadash × P2711) analyzed, 69 were resistant, while 71 lines were susceptible to PHS (Table 2). Overall, the PHS score of DH lines ranged between perfect 0 and 0.70. Almost half the lines were found susceptible to PHS with a mean score of 0.61 (0.40–0.70), and the remaining half were resistant with an average score of 0.38 (0.00–0.39), indicating, on average, a 38.8% better resistance level than that of susceptible lines.

**Figure 5:**
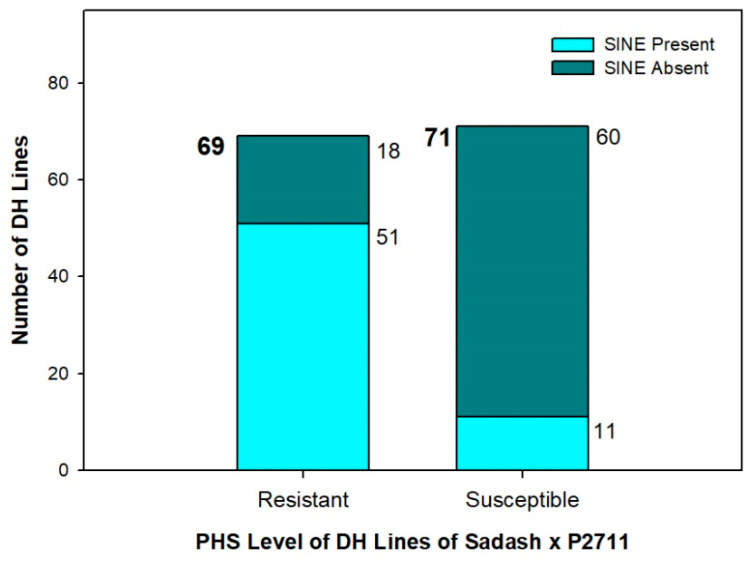
PHS level and SINE polymorphism seen in DH population (Sadash × P2711).

These lines were also evaluated for the presence of SINE insertion as P2711 and Sadash exhibit SINE polymorphism (present vs. absent, respectively). The insertion was present in 44.3% of the population (Table 2). It is interesting to note that 73.9% of resistant lines had SINE insertion, while it was absent from 85.5% of susceptible lines (Figure 5). The average PHS score of resistant lines was 0.182 (range 0.01–0.40), while it was 0.582 for susceptible lines (range 0.400–0.921). Among the resistant lines, the average PHS score was 0.303 for lines without SINE insertion, but the presence of this insertion improved the PHS resistance by 53.8% to the average PHS score of 0.140. However, no significant difference in PHS level was observed due to the presence/absence of SINE insertion among susceptible lines.

## 3. Discussion

Over the past decade, numerous studies have aimed to identify the factors influencing PHS. Despite extensive efforts, significant breakthroughs have been elusive. However, various studies have highlighted the critical role of epigenetic modification pathways in seed development [21,24]. One such pathway is RdDM, where *AGO* genes mediate the DNA methylation [25] of key genes responsible for seed development. The AGO4_9 class of genes are involved in seed development and germination, for instance, *HvAGO1003* and *HvAGO1002* in barley [22] and *TaAGO802* [23] in wheat.

Singh et al. previously identified a transposon-induced mutation (SINE) in the 3′UTR of the *AGO802B* gene in ten wheat cultivars, suggesting a potential link to PHS resistance [23]. Previous studies have highlighted the role of AGOs in various biotic and abiotic stresses [26]. In maize, the expression patterns of nine *ZmAGO* genes were significantly upregulated in response to drought [27]. Similarly, in wheat, *TaAGO2A*, 5B, 5D, 6A, 9, and 17 appeared to be affected by polyethylene glycol and salinity stresses [28]. In addition to stresses, AGO proteins are also involved in reproductive tissue development. In Arabidopsis, *AtAGO9* loaded with miRNA822 modulates monosporic female gametogenesis [29], while *OsAGO18* in rice is involved in male gametophyte development as the mutants of this gene result in stunted plants that are male-sterile [30]. Based on previous findings [22], we analyzed 41 Canadian wheat cultivars with varying levels of PHS resistance. Our findings reveal that most of the resistant cultivars (92.6%) contain this transposon insertion, whereas 64% of the susceptible cultivars lack it.

Further validation through pangenome analysis confirmed genetic variation in the *AGO802B* gene. However, many cultivars in the pangenome lacked this insertion, likely due to the pangenome cultivars being sourced from various regions globally, where this specific insertion may not be as widespread. Notably, PHS-resistant Canadian cultivar RL-4137 [31], having SINE insertion, can be traced in the pedigree of many PHS-resistant cultivars used currently. This evidence suggests that transposon insertion in *AGO802B* plays a significant role in conferring PHS resistance in the wheat cultivars under study.

Transposable elements (TEs) have the potential to generate phenotypic variation, which can facilitate rapid responses to various abiotic stresses [32]. In plants, TEs are commonly located within genes or their regulatory regions, where their position significantly affects gene expression and various regulatory mechanisms tied to growth, development, and stress adaptation [33]. An insertion of transposon-like element is known to increase the level of aluminum resistance in wheat [34]. In rice, a long terminal repeat (LTR) retrotransposon renovator is involved in the activation of the resistance gene *Pit*, which confers race-specific resistance against the fungal pathogen *Magnaporthe grisea* [35]. SINEs play a significant role in Arabidopsis; one such element is found in the promoter region of the flowering repressor gene *FWA* [36]. The epigenetic regulation of this retrotransposon helps it further modulate the gene expression. In the current study, the presence of SINE in 3′UTR enhances tolerance to PHS. 3′UTR is known to contain various motifs that control the epigenetic modulation patterns of the genes in which they are found [37]. We speculate that a regulatory mechanism similar to the one found in FWA may be at play in regulating the expression of the *AGO802* gene.

The lines in our analysis exhibited a differential expression of *TaAGO802* in relation to pre-harvest sprouting (PHS) resistance. Specifically, we observed that PHS-resistant wheat cultivars exhibit significantly lower *TaAGO802* expression, whereas susceptible cultivars demonstrate two- to fifteen-fold higher expression levels. This pattern of expression mirrors patterns previously observed in barley, for which Singh and Singh reported reduced *AGO1003* expression in resistant cultivars compared to their susceptible counterparts [22]. This conserved expression pattern across different species points to a potential role for *AGO4_9* in regulating seed dormancy and germination. Meng et al. discovered that the expression of *TaAGO4* was greatly downregulated in the endosperm tissues during seed imbibition, and this coincided with the upregulation of enzymes during germination [38].

Multiple studies on major methylases have underscored the correlation between methylation and seed development in wheat [39], rice [40], soybean [41], and Arabidopsis [42]. In our study, the global methylation (5-mC%) analysis revealed that the methylation level of the PHS-resistant cultivars such as RL4137, Enchant, and Stettler was significantly lower than those of the susceptible cultivars such as AAC Innova, AC Andrew, and AAC Awesome. Similar results were obtained in *Brassica napus*, where in response to heat stress, the resistant genotype was hypomethylated compared to the susceptible ones [43]. In rice, hypomethylation is commonly observed in several stress-responsive genes and transposable elements [44]. Interestingly, the resistant cultivar AAC Tenacious showed higher expression of *TaAGO802* and global methylation levels compared to other resistant cultivars, likely due to the absence of SINE insertion at the 3′UTR of *TaAGO802B*. These results suggest that resistance in AAC Tenacious may also be regulated by the presence of other pathway genes, such as *MFT-A1b*, *MFT-3B-1*, *HUB1*, *TaVp1-D1*, and *TaMyb10-D1*, which are involved in embryo- and seed coat-imposed dormancy, as previously reported [6]. Further studies could provide a more comprehensive understanding of the underlying associations between SINE insertion and the PHS response. This is particularly important in light of the fact that even established tests like falling numbers cannot accurately and certainly predict PHS response [45].

In line with the presence of SINE insertion and DNA methylation polymorphisms on the cultivar panel, the DH population exhibited similar patterns. The SINE was seen in 3/4th s of resistant lines, while nearly 85% of the susceptible lines lacked the insertion. In general, the SINE insertion increased the tolerance level of wheat lines/cultivars to PHS. This could be mediated by decreasing the expression of the *TaAGO802* gene, as seen currently. This decrease in the availability of AGO could in turn reduce the formation of the siRNA-AGO complex, which would then lower the DNA methylation levels in these resistant cultivars. In plants, cytosine methylation is a conserved epigenetic mark critical for the regulation of gene expression [46]. This mark is distributed, among other regions, in repetitive DNA and TEs like SINE insertion. When present at gene regulatory sites such as UTRs and promoters, DNA methylation can trigger transcriptional gene silencing (TGS) [47]. Interestingly, CG methylation is often found within the gene bodies of housekeeping or constitutively expressed genes in many plant species, though its precise functional role in these contexts remains uncertain [46]. The reduced DNA methylation level might allow the dormancy-promoting genes such as *TaMFT*, *TaMKK*, and *TaSdr* to express, and this would increase the level of seed dormancy in the resistant cultivars as well as lead to increased PHS tolerance levels.

## 4. Materials and Methods

### 4.1. Plant Material and Spike Collection

A set of 41 Canadian wheat cultivars, representing various levels of PHS tolerance, were employed for analysis. Of these, twenty-three cultivars were grown in a greenhouse under controlled conditions, with daytime temperatures maintained at 22 °C for 16 h and nighttime temperatures at 18 °C for 8 h. A 20:20:20 fertilizer (nitrogen–phosphorus–potassium) was applied both after sowing and during the tillering stage to promote plant growth. At the flowering stage, spikes were tagged on the day of pollination (identified by the appearance of yellow anthers after plucking a spikelet from the center of the spike). Two spikes from two different plants representing two biological replicates were sampled from a subset of twenty-three cultivars at 20 days after pollination (DAP) for RNA isolation. The spikes were immediately frozen in liquid nitrogen and stored at −80 °C until further use. Sampling was also carried out upon seed maturation, wherein 15–20 seeds from each cultivar were stored at −80 °C for DNA isolation.

Additionally, a doubled haploid (DH) population was developed by crossing Sadash, used as a female parent with P2711 as a male parent. F_1_s produced from ‘Sadash/P2711’ crosses were used for doubled haploid production. A total of 140 DH lines produced from this cross were employed for further analysis. Sadash is a soft white spring wheat cultivar that is susceptible to PHS and lacks SINE insertion, whereas P2711 is an experimental spring wheat breeding line from South Africa that is resistant to PHS and also possesses a SINE insertion. Common wheat cvs. Lilian, Carberry, Brandon, Chiffon, Innova, Indus, and CDC Stanley were used as checks for comparisons.

### 4.2. Genomic DNA Isolation

For each cultivar, 2–3 mature seeds were surface-sterilized with 20% bleach and thoroughly rinsed with water. The seeds were then placed on filter paper in Petri plates for germination. After two weeks, young leaves from each cultivar were collected. DNA was extracted using the Urea method [48] and quantified using a NanoDrop ND-1000 (NanoDrop Technologies, Wilmington, DE, USA). The integrity and purity of the genomic DNA was assessed with 0.8% agarose gel electrophoresis. Additionally, DNA was isolated from mature seeds, which were cut in half, ensuring that one half contained intact embryos [49,50].

### 4.3. PCR and Determination of SINE Polymorphism

To ascertain the presence or absence of SINE in wheat cultivars, PCR amplification was performed using specific primers (Supplementary Table S2) followed by gel electrophoresis. The resultant bands were eluted from the gel and outsourced for Sanger sequencing to Genome Quebec. The sequences thus obtained were analyzed using a multiple alignment tool—Multialin [51]. Publicly available pangenome data were also used for the in silico analysis of the wheat *AGO802* gene [52,53].

### 4.4. RNA Isolation and cDNA Synthesis

The immature embryos were carefully isolated from the medial seeds of previously stored 20DAP wheat spikes into 1.5 mL vials. The immature embryos were chilled in liquid nitrogen and then crushed with pellet pestles. Total RNA was extracted using Sigma-Aldrich’s Spectrum^TM^ Plant Total RNA Kit per the manufacturer’s protocol. RNA was then quantified using the NanoDrop ND-1000 (NanoDrop Technologies, Wilmington, DE, USA). The RNA was then treated with DNase (Invitrogen, Burlington, ON, Canada) to eliminate DNA contamination; 500 ng of pure RNA thus obtained was utilized for cDNA synthesis using the AffinityScript QPCR cDNA Synthesis Kit (Agilent Technologies Canada Inc., Mississauga, ON, Canada). The integrity of the cDNA was validated through standard RT-PCR with wheat-specific ACTIN primers (Supplementary Table S1) followed by gel electrophoresis.

### 4.5. qRT-PCR Analysis

qRT-PCR was carried out on three technical replicates each of the cDNA obtained from two biological replicates per cultivar along with two replicates of the no-template control. The qRT-PCR was executed on an Mx3005 instrument (Agilent Technologies Canada Inc., Mississauga, ON, Canada) using Wisent low Rox Supergreen QPCR master mix (Agilent Technologies Canada Inc., Mississauga, ON, Canada) per the manufacturer’s protocol. A 20 µL reaction mixture was set up for amplification, containing 10µM of both forward and reverse gene-specific primers (qRT802-FP and qRT802-RP) (Supplementary Table S1), 1X Wisent low Rox Supergreen QPCR master mix, and 1 µL (500 ng) of cDNA template. Thermocycling was carried out by running the reaction at 95 °C for 2 min, followed by 40 cycles of denaturation at 95 °C for 15 s and 60 °C for 30 s. Internal controls included the expression of a reference gene *ACTIN*, a housekeeping gene in wheat. The relative expression of the gene was analyzed using the 2^−ΔΔCq^ method [54].

### 4.6. DNA Methylation Analysis

The global DNA methylation status of contrasting wheat genotypes was estimated using the Abcam Global Methylation Assay Kit. Both positive and negative DNA controls provided in the kit were included to establish a standard curve for 5-mC quantification. Genomic DNA (100 ng) extracted from mature seeds was utilized for the detection of 5-methylcytosine (5-mC) using the manufacturer’s protocol. Genomic DNA samples were coated in triplicate onto the assay plate wells. Absorbance was recorded at 450 nm after color development. The percentage of 5-mC was quantified by plotting the standard curve and applying the specified formula [55]:

$$5-mC\%=\frac{Sample\;OD-Negative\;Control\;OD}{Slope\times S}\times 100\%$$

where S is the amount of input sample DNA in ng.

### 4.7. PHS Phenotyping

PHS tolerance was assayed using a protocol modified from the method proposed by Hisano et al. [56]. In total, 15 physiologically mature spike heads (3 biological reps of 5 spikes each) of each genotype were collected and allowed to dry at room temperature for 2 days. Subsequently, the harvested spikes were immersed in water for 5 min. and then laid horizontally on trays, which were covered with domes to maintain high humidity. The trays were placed in growth chamber maintained with a 16 h light period at 25 °C and 8 h dark period at 15 °C. Wet conditions were maintained by spraying the spikes with water thrice daily. Germinated grains were counted daily, and the results were expressed as either a cumulative percentage sprouting curve or a weighted sprouting index [57]. The maximum index is 1 if all grains sprouted by day 1, whilst lower indices are indicative of increasing levels of grain dormancy or PHS resistance. The PHS score of each cultivar was calculated by averaging the PHS score of three replicates, which was further averaged from the five spikes comprising that replicate.

### 4.8. Statistical Analysis

One-way analysis of variance (ANOVA) and Tukey’s test (*p* ≤ 0.05) were carried out using JMP Pro 17 software to determine statistical significance in the *TaAGO802* expression and 5-mC% methylation level among different wheat cultivars with varied PHS responses.

## 5. Conclusions

PHS is a complex trait influenced by numerous genetic and environmental factors. Our findings highlight the critical role of epigenetic regulation, particularly the influence of a transposon-induced mutation in the *AGO802B* gene, in controlling seed dormancy and PHS resistance. The SINE within *AGO802B* offers potential as a genetic marker for identifying PHS-resistant wheat cultivars, especially within Canadian breeding programs. These insights provide a valuable tool for the development of PHS-resistant wheat cultivars, contributing to global food security in the face of climate change and other environmental challenges. By leveraging genetic and epigenetic markers, breeders can better select traits that enhance crop resilience, ensuring stable yields under increasingly variable environmental conditions.

## Figures and Tables

**Table 1:** List of wheat cultivars displaying variation in SINE insertion.

PHS Response	SINE-Present	SINE-Absent
Resistant	CDC Kernen, AAC Goodwin, Glenn, AAC Westlock, SC-8021-V2, HY2096, BW5070, BW5077, HY2122, HY2135, Snowbird, HY2152, AAC Camrose, HY2141, Enchant, Cardale, AAC Penhold, Carberry, RL4137, RL4555, Stettler, CDC Stanley, Vesper, Lilian, P2711	Pasteur, AAC Tenacious
Susceptible	Fielder, AAC Iceberg, AAC Whitehead, AAC Foray, AAC Brandon	Conquer, HY2120, HY2136, Sadash, AAC Chiffon, AAC Innova, AAC Indus, AAC Awesome, AC Andrew

**Table 2:** Association of PHS score and SINE insertion in DH lines of Sadash (Susceptible) × P2711 (resistant) population.

Population Subset	No. of Cultivars	No. of Cultivars with SINE Insertion	Mean PHS Score (Range)
Resistant	69	51	0.182 (0.01–0.40)
Susceptible	71	11	0.582 (0.40–0.92)
Total	140	62	

## Data Availability

Data are contained within the article.

## Supplementary Materials

The following supporting information can be downloaded at: https://www.mdpi.com/article/10.3390/plants13212981/s1, Table S1: List of wheat pangenome varieties showing differences in SINE insertion in *AGO802* gene; Table S2: List of primers used in the study; Figure S1: Multiple sequence alignment of pangenome data from various wheat varieties.

## References

[1] Food and Agriculture Organization of the United Nations FAOStat. http://www.fao.org/faostat.

[2] Senapati N., Semenov M.A. (2020). Large genetic yield potential and genetic yield gap estimated for wheat in Europe. Glob. Food Secur..

[3] Mao H., Jiang C., Tang C., Nie X., Du L., Liu Y., Cheng P., Wu Y., Liu H., Kang Z. (2023). Wheat adaptation to environmental stresses under climate change: Molecular basis and genetic improvement. Mol. Plant.

[4] Gupta P.K., Balyan H.S., Sharma S., Kumar R. (2020). Genetics of yield, abiotic stress tolerance and biofortification in wheat (*Triticum aestivum* L.). Theor. Appl. Genet..

[5] Chang C., Zhang H., Lu J., Si H., Ma C. (2023). Genetic improvement of wheat with pre-harvest sprouting resistance in China. Genes.

[6] Dhariwal R., Hiebert C.W., Sorrells M.E., Spaner D., Graf R.J., Singh J., Randhawa H.S. (2021). Mapping pre-harvest sprouting resistance loci in AAC Innova× AAC Tenacious spring wheat population. BMC Genom..

[7] Matilla A.J. (2022). Exploring breakthroughs in three traits belonging to seed life. Plants.

[8] Derera N. (2018). The effects of preharvest rain. Preharvest Field Sprouting in Cereals.

[9] Rodríguez M.V., Barrero J.M., Corbineau F., Gubler F., Benech-Arnold R.L. (2015). Dormancy in cereals (not too much, not so little): About the mechanisms behind this trait. Seed Sci. Res..

[10] Patwa N., Penning B.W., Roychowdhury R., Choudhury S., Hasanuzzaman M., Srivastava S. (2020). Environmental impact on cereal crop grain damage from pre-harvest sprouting and late maturity alpha-amylase. Sustainable Agriculture in the Era of Climate Change.

[11] Ceccato D.V., Bertero H.D., Batlla D. (2011). Environmental control of dormancy in quinoa (*Chenopodium quinoa*) seeds: Two potential genetic resources for pre-harvest sprouting tolerance. Seed Sci. Res..

[12] Vetch J.M., Stougaard R.N., Martin J.M., Giroux M.J. (2019). Revealing the genetic mechanisms of pre-harvest sprouting in hexaploid wheat (*Triticum aestivum* L.). Plant Sci..

[13] Singh C., Kamble U.R., Gupta V., Singh G., Sheoran S., Gupta A., Tyagi B.S., Kumar P., Mishra C.N., Krishannapa G. (2021). Pre-harvest sprouting in wheat: Current status and future prospects. J. Cereal Res..

[14] Duffus C. (2019). Recent progress in the physiology and biochemistry of immature cereal grains in relation to pre-harvest sprouting. Fourth International Symposium on Pre-Harvest Sprouting in Cereals.

[15] Zhou G., Wu S., Chen D., Wu X., Cai Q. (2023). Polyphenols and phytohormones profiling of pre-harvest sprouting resistant and susceptible wheat genotypes. SN Appl. Sci..

[16] Tai L., Wang H.-J., Xu X.-J., Sun W.-H., Ju L., Liu W.-T., Li W.-Q., Sun J., Chen K.-M. (2021). Pre-harvest sprouting in cereals: Genetic and biochemical mechanisms. J. Exp. Bot..

[17] Ali A., Cao J., Jiang H., Chang C., Zhang H.-P., Sheikh S.W., Shah L., Ma C. (2019). Unraveling molecular and genetic studies of wheat (Triticum aestivum L.) resistance against factors causing pre-harvest sprouting. Agronomy.

[18] Yiwen H., Xuran D., Hongwei L., Shuo Y., Chunyan M., Liqiang Y., Guangjun Y., Li Y., Yang Z., Hongjie L. (2022). Identification of effective alleles and haplotypes conferring pre-harvest sprouting resistance in winter wheat cultivars. BMC Plant Biol..

[19] Schulthess A.W., Reif J.C., Ling J., Plieske J., Kollers S., Ebmeyer E., Korzun V., Argillier O., Stiewe G., Ganal M.W. (2017). The roles of pleiotropy and close linkage as revealed by association mapping of yield and correlated traits of wheat (*Triticum aestivum* L.). J. Exp. Bot..

[20] Nonogaki H. (2014). Seed dormancy and germination—Emerging mechanisms and new hypotheses. Front. Plant Sci..

[21] Ding X., Jia X., Xiang Y., Jiang W. (2022). Histone modification and chromatin remodeling during the seed life cycle. Front. Plant Sci..

[22] Singh M., Singh J. (2012). Seed development-related expression of ARGONAUTE4_9 class of genes in barley: Possible role in seed dormancy. Euphytica.

[23] Singh M., Singh S., Randhawa H., Singh J. (2013). Polymorphic homoeolog of key gene of RdDM pathway, ARGONAUTE4_9 class is associated with pre-harvest sprouting in wheat (*Triticum aestivum* L.). PLoS ONE.

[24] Liton M.U.A., McCartney C.A., Hiebert C.W., Kumar S., Jordan M.C., Ayele B.T. (2021). Identification of loci for pre-harvest sprouting resistance in the highly dormant spring wheat RL4137. Theor. Appl. Genet..

[25] Erdmann R.M., Picard C.L. (2020). RNA-directed DNA methylation. PLoS Genet..

[26] Zaheer U., Munir F., Salum Y.M., He W. (2024). Function and regulation of plant ARGONAUTE proteins in response to environmental challenges: A review. PeerJ.

[27] Zhai L., Teng F., Zheng K., Xiao J., Deng W., Sun W. (2019). Expression analysis of Argonaute genes in maize (*Zea mays* L.) in response to abiotic stress. Hereditas.

[28] Liu Y.-F., Wang L.-M., Zhao L.-Z., Wang W., Zhang H.-X. (2021). Genome-Wide Identification and Evolutionary Analysis of Argonaute Genes in Hexaploid Bread Wheat. BioMed Res. Int..

[29] Tovar-Aguilar A., Grimanelli D., Acosta-García G., Vielle-Calzada J.-P., Badillo-Corona J.A., Durán-Figueroa N. (2024). The miRNA822 loaded by ARGONAUTE9 modulates the monosporic female gametogenesis in *Arabidopsis thaliana*. Plant Reprod..

[30] Das S., Swetha C., Pachamuthu K., Nair A., Shivaprasad P. (2020). Loss of function of *Oryza sativa* Argonaute 18 induces male sterility and reduction in phased small RNAs. Plant Reprod..

[31] DePauw R., Clarke F., Fofana B., Knox R., Humphreys G., Cloutier S. (2009). RL4137 contributes preharvest sprouting resistance to Canadian wheats. Euphytica.

[32] Ezin V., Symonds R.C. (2023). Role of Transposable Elements in Plants Under Abiotic Stress Response. Plant Transposable Elements.

[33] Ramakrishnan M., Satish L., Kalendar R., Narayanan M., Kandasamy S., Sharma A., Emamverdian A., Wei Q., Zhou M. (2021). The dynamism of transposon methylation for plant development and stress adaptation. Int. J. Mol. Sci..

[34] Tovkach A., Ryan P.R., Richardson A.E., Lewis D.C., Rathjen T.M., Ramesh S., Tyerman S.D., Delhaize E. (2013). Transposon-mediated alteration of TaMATE1B expression in wheat confers constitutive citrate efflux from root apices. Plant Physiol..

[35] Hayashi K., Yoshida H. (2009). Refunctionalization of the ancient rice blast disease resistance gene Pit by the recruitment of a retrotransposon as a promoter. Plant J..

[36] Kinoshita Y., Saze H., Kinoshita T., Miura A., Soppe W.J., Koornneef M., Kakutani T. (2007). Control of FWA gene silencing in Arabidopsis thaliana by SINE-related direct repeats. Plant J..

[37] Hardy E.C., Balcerowicz M. (2024). Untranslated yet indispensable—UTRs act as key regulators in the environmental control of gene expression. J. Exp. Bot..

[38] Meng F., Jia H., Ling N., Xue Y., Liu H., Wang K., Yin J., Li Y. (2013). Cloning and characterization of two Argonaute genes in wheat (*Triticum aestivum* L.). BMC Plant Biol..

[39] Dai Y., Ni Z., Dai J., Zhao T., Sun Q. (2005). Isolation and expression analysis of genes encoding DNA methyltransferase in wheat (*Triticum aestivum* L.). Biochim. Biophys. Acta (BBA) Gene Struct. Expr..

[40] Hu L., Li N., Xu C., Zhong S., Lin X., Yang J., Zhou T., Yuliang A., Wu Y., Chen Y.-R. (2014). Mutation of a major CG methylase in rice causes genome-wide hypomethylation, dysregulated genome expression, and seedling lethality. Proc. Natl. Acad. Sci. USA.

[41] Song Q.-X., Lu X., Li Q.-T., Chen H., Hu X.-Y., Ma B., Zhang W.-K., Chen S.-Y., Zhang J.-S. (2013). Genome-wide analysis of DNA methylation in soybean. Mol. Plant.

[42] Hsieh T.-F., Ibarra C.A., Silva P., Zemach A., Eshed-Williams L., Fischer R.L., Zilberman D. (2009). Genome-wide demethylation of Arabidopsis endosperm. Science.

[43] Gao G., Li J., Li H., Li F., Xu K., Yan G., Chen B., Qiao J., Wu X. (2014). Comparison of the heat stress induced variations in DNA methylation between heat-tolerant and heat-sensitive rapeseed seedlings. Breed. Sci..

[44] Arora H., Singh R.K., Sharma S., Sharma N., Panchal A., Das T., Prasad A., Prasad M. (2022). DNA methylation dynamics in response to abiotic and pathogen stress in plants. Plant Cell Rep..

[45] Hu Y., Sjoberg S.M., Chen C., Hauvermale A.L., Morris C.F., Delwiche S.R., Cannon A.E., Steber C.M., Zhang Z. (2022). As the number falls, alternatives to the Hagberg–Perten falling number method: A review. Compr. Rev. Food Sci. Food Saf..

[46] Luján-Soto E., Dinkova T.D. (2021). Time to wake up: Epigenetic and small-RNA-mediated regulation during seed germination. Plants.

[47] El-Sappah A.H., Yan K., Huang Q., Islam M.M., Li Q., Wang Y., Khan M.S., Zhao X., Mir R.R., Li J. (2021). Comprehensive mechanism of gene silencing and its role in plant growth and development. Front. Plant Sci..

[48] Nalini E., Bhagwat S., Jawali N. (2004). A simple method for isolation of DNA from plants suitable for long term storage and DNA marker analysis. BARC Newsl..

[49] Júnior C.D.S., Teles N.M.M., Luiz D.P., Isabel T.F., Micic M. (2016). DNA extraction from seeds. Sample Preparation Techniques for Soil, Plant, and Animal Samples.

[50] GeneLink DNA & RNA Precipitation Solutions. https://www.genelink.com/Literature/ps/PS40-5132_PptionSoln_Ver3.3.pdf.

[51] Corpet F. (1988). Multiple sequence alignment with hierarchical clustering. Nucleic Acids Res..

[52] Montenegro J.D., Golicz A.A., Bayer P.E., Hurgobin B., Lee H., Chan C.K.K., Visendi P., Lai K., Doležel J., Batley J. (2017). The pangenome of hexaploid bread wheat. Plant J..

[53] Yates A.D., Allen J., Amode R.M., Azov A.G., Barba M., Becerra A., Bhai J., Campbell L.I., Carbajo Martinez M., Chakiachvili M. (2022). Ensembl Genomes 2022: An expanding genome resource for non-vertebrates. Nucleic Acids Res..

[54] Livak K.J., Schmittgen T.D. (2001). Analysis of relative gene expression data using real-time quantitative PCR and the 2^−ΔΔCT^ method. Methods.

[55] Abcam Global DNA Methylation Assay Kit (5 Methyl Cytosine, Colorimetric). https://www.abcam.com/en-ca/products/assay-kits/global-dna-methylation-assay-kit-5-methyl-cytosine-colorimetric-ab233486#tab=support.

[56] Hisano H., Hoffie R.E., Abe F., Munemori H., Matsuura T., Endo M., Mikami M., Nakamura S., Kumlehn J., Sato K. (2022). Regulation of germination by targeted mutagenesis of grain dormancy genes in barley. Plant Biotechnol. J..

[57] Walker-Simmons M. (1988). Enhancement of ABA responsiveness in wheat embryos by high temperature. Plant Cell Environ..

