# Supplementary material for: Utilizing Short Interspersed Nuclear Element as a Genetic Marker for Pre-Harvest Sprouting in Wheat
Source: Plants (Basel). 2024 Oct 25;13(21):2981. doi: 10.3390/plants13212981 (PMC11548262; doi:10.3390/plants13212981)
Supplement: Supplementary file 1 [file plants-13-02981-s001.zip › plants-3243504-supplementary.pdf]

Supplementary Table S1. List of wheat pangenome varieties showing differences in SINE-insertion in *AGO802* gene.

**Varieties without SINE-insertion**	**Varieties with SINE-insertion**
Arinalrfor, Chinese Spring, Jagger, Julius, Norin 61, Mace, Sy Mattis, Cadenza, Paragon, Robigus, Claire, Weebill, Renan	Kariega, Lancer, Landmark

Supplementary Table S2. List of primers used in the study

S.No.	Primer ID	Primer sequence (5′ → 3′)	Purpose
**1.**	37533F1	GACTACAAGGCCAACGCACT	Wheat SINE study
**2.**	37533R1	AAGGTTCAACAGCAGCATCC	Wheat SINE study
**3.**	*TaACTIN*-F	ACCTTCAGTTGCCCAGCAAT	Wheat ACTIN
**4.**	*TaACTIN*-R	CAGAGTCGAGCACAATACCAGTTG	Wheat ACTIN
**5.**	qRT802-FP	AGAAGCCATTCGGGTTATTG	Wheat qRT primers
**6.**	qRT802-RP	TTAGGGTGGTCAACCTTCTG	Wheat qRT primers

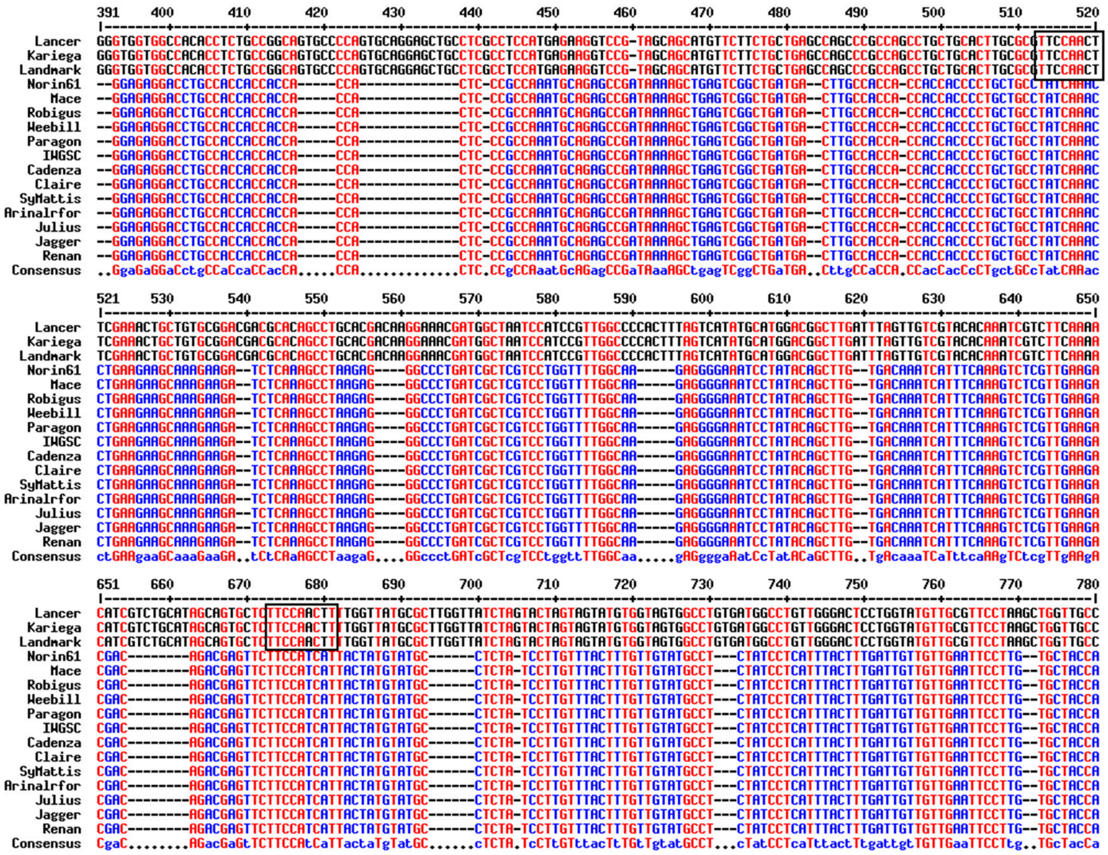

Supplementary Figure S1. Multiple sequence alignment of pangenome data from various wheat varieties. The black boxes indicate the 9-bp repeats of the SINE insertion.
